# ADVANCING POLICY TO BUILD A BEHAVIORAL HEALTH WORKFORCE THAT ADDRESSES THE NEEDS OF OLDER ADULTS

**DOI:** 10.1093/geroni/igz038.2462

**Published:** 2019-11-08

**Authors:** Kathryn G Kietzman, Alina Palimaru, Janet C Frank

**Affiliations:** 1 UCLA Center for Health Policy Research, Los Angeles, California, United States; 2 RAND Corporation, Santa Monica, California, United States

## Abstract

California’s Mental Health Services Act has infused funding for workforce education and training into the public mental health system. However, funding has not kept pace with an existing behavioral health workforce shortage crisis, the rapid growth of an aging population, and the historical lack of geriatric training in higher education for the helping professions. This study draws on findings from a recent evaluation of how older adults are served by California’s public mental health delivery system, and a review of state planning documents and academic literature, to describe gaps and deficiencies in the workforce that serves older adults. While California has more than 80,000 licensed behavioral health professionals in a variety of disciplines, very few have specialized training in geriatrics. Across the U.S., there are fewer than 1,800 geriatric psychiatrists and only about 3% of medical students take any geriatrics electives during their training. Very few nurses (1%), psychologists (4%), or social workers (4%) have training in and/or specialize in geriatrics. Of additional concern in California is the lack of representation of ethnic and racial minorities, and rural/urban geographic disparities in the distribution of the behavioral health workforce. Recommendations for advancing policy change to improve the preparation and distribution of the geriatric behavioral workforce are presented to three distinct audiences: state policymakers and administrators; educational institutions, accrediting bodies, and licensing boards; and county mental health/behavioral health departments and their contracted providers.

